# Inhibitory Effect of Isopanduratin A on Adipogenesis: A Study of Possible Mechanisms

**DOI:** 10.3390/foods12051014

**Published:** 2023-02-27

**Authors:** Prapenpuksiri Rungsa, Htoo Tint San, Boonchoo Sritularak, Chotima Böttcher, Eakachai Prompetchara, Chatchai Chaotham, Kittisak Likhitwitayawuid

**Affiliations:** 1 Department of Pharmacognosy and Pharmaceutical Botany, Faculty of Pharmaceutical Sciences, Chulalongkorn University, Bangkok 10330, Thailand; 2 Center of Excellence in Natural Products for Ageing and Chronic Diseases, Faculty of Pharmaceutical Sciences, Chulalongkorn University, Bangkok 10330, Thailand; 3 Experimental and Clinical Research Center, a Cooperation between the Max Delbrück Center for Molecular Medicine in the Helmholtz Association and Charité–Universitätsmedizin Berlin, 13125 Berlin, Germany; 4 Max Delbrück Center for Molecular Medicine in the Helmholtz Association (MDC), 13125 Berlin, Germany; 5 Department of Laboratory Medicine, Faculty of Medicine, Chulalongkorn University, Bangkok 10330, Thailand; 6 Center of Excellence in Vaccine Research and Development (Chula Vaccine Research Center), Faculty of Medicine, Chulalongkorn University, Bangkok 10330, Thailand; 7 Department of Biochemistry and Microbiology, Faculty of Pharmaceutical Sciences, Chulalongkorn University, Bangkok 10330, Thailand; 8 Preclinical Toxicity and Efficacy Assessment of Medicines and Chemicals Research Unit, Faculty of Pharmaceutical Sciences, Chulalongkorn University, Bangkok 10330, Thailand

**Keywords:** fingerroot, *Boesenbergia rotunda*, obesity, adipocyte, isopanduratin A, AKT/GSK3β, AMPK-ACC, MAPKs, MCE

## Abstract

The root of *Boesenbergia rotunda*, a culinary plant commonly known as fingerroot, has previously been reported to possess anti-obesity activity, with four flavonoids identified as active principles, including pinostrobin, panduratin A, cardamonin, and isopanduratin A. However, the molecular mechanisms underlying the antiadipogenic potential of isopanduratin A remain unknown. In this study, isopanduratin A at non-cytotoxic concentrations (1–10 μM) significantly suppressed lipid accumulation in murine (3T3-L1) and human (PCS-210-010) adipocytes in a dose-dependent manner. Downregulation of adipogenic effectors (FAS, PLIN1, LPL, and adiponectin) and adipogenic transcription factors (SREBP-1c, PPARγ, and C/EBPα) occurred in differentiated 3T3-L1 cells treated with varying concentrations of isopanduratin A. The compound deactivated the upstream regulatory signals of AKT/GSK3β and MAPKs (ERK, JNK, and p38) but stimulated the AMPK-ACC pathway. The inhibitory trend of isopanduratin A was also observed with the proliferation of 3T3-L1 cells. The compound also paused the passage of 3T3-L1 cells by inducing cell cycle arrest at the G0/G1 phase, supported by altered levels of cyclins D1 and D3 and CDK2. Impaired p-ERK/ERK signaling might be responsible for the delay in mitotic clonal expansion. These findings revealed that isopanduratin A is a strong adipogenic suppressor with multi-target mechanisms and contributes significantly to anti-obesogenic activity. These results suggest the potential of fingerroot as a functional food for weight control and obesity prevention.

## 1. Introduction

With the steady increase in the number of overweight and obese populations in recent years, obesity has been declared a pandemic disease by the World Health Organization (WHO) [1]. Obesity is the result of an energy imbalance, characterized by excessive fat accumulation in the body. This irregularity, though a non-communicable disorder, is closely associated with several metabolic conditions, such as hyperglycemia, hyperlipidemia, hypertension, cancer, and cardiovascular diseases, all of which have a high mortality rate and can cause a socioeconomic burden, particularly in countries where access to the healthcare system is limited [2].

Modulation of the excess mass of adipose tissues due to hyperplasia (excessive adipogenesis) and the hypertrophy of adipocytes is one of the reasonable strategies to regulate lipid homeostasis and obesity. Recently, inhibition of adipogenic differentiation and maturation has become a novel therapeutic approach to treating obesity [3]. Adipogenesis, a multistep process that converts undifferentiated preadipocytes into mature adipocytes, is modulated by a series of biochemical cascades that include coordinated changes in hormone sensitivity and gene expression, together with morphological alterations. Triggered by adipogenic stimulants, preadipocytes undergo mitotic clonal expansion (MCE) to re-enter the cell cycle. Concurrently, the upregulation of adipogenic regulating genes and adipogenic effector proteins leads to adipocyte differentiation and maturation [4,5,6,7].

Adipocyte differentiation and development are directed by lipogenesis-related transcription factors such as CCAAT/enhancer-binding protein alpha (C/EBPα), peroxisome proliferator-activated receptor gamma (PPARγ), sterol response element-binding protein-1c (SREBP-1c) [8,9], and the adenosine monophosphate-activated protein kinase (AMPK) and acetyl-CoA carboxylase (ACC) enzymes [10]. AMPK, a serine/threonine kinase, forms a heterotrimeric complex with one catalytic α subunit and two regulatory β and γ subunits [11]. Its roles in cellular lipid metabolism involve the synthesis and degradation of fatty acids. Another upstream regulatory molecule in adipocyte differentiation is protein kinase B (AKT), as its activation strongly links to the upregulation of SREBP-1c and cellular lipogenesis [12]. Subsequent phosphorylation of glycogen synthase kinase 3β (GSK3β) by AKT upregulates C/EBPα and promotes adipocyte maturation [13]. Additionally, mitogen-activated protein kinases (MAPKs), including c-Jun N-terminal kinase (JNK), extracellular signal-regulated kinase (ERK), and stress-activated protein kinase (p38), mediate adipogenesis [14]. Suppression of these signaling molecules efficiently inhibits adipocyte differentiation [15,16]. For example, inhibition of p38 function can hamper adipocyte differentiation by suppressing PPARγ transcription. Modulation of these biomolecules during adipocyte differentiation proved to be a promising strategy to limit cellular lipogenesis and adipocyte differentiation and maturation [17].

Recently, a growing body of evidence has revealed medicinal and culinary plants as a rich source of phytochemicals that exert their anti-obesity potential through multi-target mechanisms [18,19,20]. *Boesenbergia rotunda* (L.) Mansf., also known as *Boesenbergia pandurata* (Roxb.) Schltr., is commonly called fingerroot. The plant is found in the wild and is widely cultivated in South Asia and Southeast Asia [21,22]. Traditionally, people use its roots as food and flavoring agents. In Thailand, they are the main ingredient in shrimp soup, which is popularly consumed by lactating women to help improve their breast milk supply. Various medicinal values for fingerroot were reported, including anti-inflammatory, antimicrobial, antiviral [21,22,23,24], anti-obesity [25], anti-osteoporosis [26], and anticancer activities [27], as well as aphrodisiac and vasorelaxant effects [28]. The bioactive constituents were characterized as several subclasses of flavonoids [29,30].

In a recent study, the anti-obesity activity of fingerroot was demonstrated in mice on a high-fat diet [31]. Our previous phytochemical study of the roots of this plant revealed the presence of several flavonoids, along with a monoterpene alcohol and a styrylpyrone [32]. In a preliminary Oil Red O assay, we found that the flavonoids pinostrobin, panduratin A, isopanduratin A, and cardamonin were strong adipogenic inhibitors, which may be responsible for the anti-obesity activity of fingerroot (see Section 3.1). In our previous study, pinostrobin was shown to inhibit adipogenesis in murine 3T3-L1 preadipocytes by lowering the levels of lipid-metabolism-mediating proteins, such as C/EBPα, PPARγ, and SREBP-1c, and suppressing the signals of MAPKs (p38 and JNK) and AKT (AKT/GSK3β and AKT/AMPKα-ACC) [33]. The other flavonoids, i.e., panduratin A and cardamonin, were previously investigated for the molecular mechanisms underlying their anti-adipogenic effects in 3T3-L1 cells [25,34,35]. In this study, we report the inhibitory effects of isopanduratin A, another fingerroot flavonoid, on adipogenesis in mouse 3T3-L1 and human PCS-210-010 preadipocytes. The relevant molecular mechanisms are also elucidated and addressed.

## 2. Materials and Methods

### 2.1. Chemicals, Reagents, and Culture Media

Isopanduratin A and other phytochemicals were isolated and characterized from *B. rotunda* roots with a protocol described previously [32]. The purity of these phytochemicals was more than 98% (by NMR). Dimethyl sulfoxide (DMSO), Oil Red O, crystal violet, isobutylmethylxanthine (IBMX), dexamethasone, isopropanol, RNase A, and skim milk powder were purchased from Sigma-Aldrich (St. Louis, MO, USA). Ethanol, methanol, formaldehyde, and chloroform were ordered from Merck KgaA (Darmstadt, Germany). Dulbecco’s Modified Eagle Medium (DMEM), fetal bovine serum (FBS), penicillin/streptomycin solution, l-glutamine, and trypsin were bought from Gibco (Gaithersburg, MA, USA). Fibroblast basal medium (FBM) was purchased from the American Type Culture Collection (ATCC; Manassas, VA, USA). Insulin was ordered from Himedia (Mumbai, India). Bicinchoninic acid (BCA) protein assay kit, western chemiluminescent ECL substrate, and radio-immunoprecipitation assay (RIPA) buffer were acquired from Thermo-Fisher (Rockford, IL, USA). A protease inhibitor cocktail was obtained from Roche Applied Science (Indianapolis, IN, USA). Primary antibodies against β-actin (Cat. No. 4970; dilution 1:1000), Cyclin D1 (Cat. No. 2978; dilution 1:1000), Cyclin D3 (Cat. No. 2936; dilution 1:2000), CDK2 (Cat. No. 2546; dilution 1:1000), AKT (Cat. No. 4691; dilution 1:1000), p-AKT (Ser473) (Cat. No. 4060; dilution 1:2000), GSK3β (Cat. No. 12456; dilution 1:1000), p-GSK3β (Ser9) (Cat. No. 9322; dilution 1:1000), AMPKα (Cat. No. 5831; dilution 1:1000), p-AMPKα (Thr172) (Cat. No. 2535; dilution 1:1000), AMPKβ1/2 (Cat. No. 4150; dilution 1:1000), p-AMPKβ1 (Ser182) (Cat. No. 4186; dilution 1:1000), ACC (Cat. No. 3676; dilution 1:1000), p-ACC (Ser79) (Cat. No. 11818; dilution 1:1000), PPARγ (Cat. No. 2435; dilution 1:1000), C/EBPα (Cat. No. 8178; dilution 1:1000), FAS (Cat. No. 3180; dilution 1:1000), PLIN1 (Cat. No. 9349; dilution 1:1000), adiponectin (Cat. No. 2789; dilution 1:1000), ERK1/2 (Cat. No. 9102; dilution 1:1000), p-ERK1/2 (Thr202/Tyr204) (Cat. No. 4695; dilution 1:1000), JNK (Cat. No. 9252; dilution 1:1000), p-JNK (Thr183/Tyr185) (Cat. No. 9251; dilution 1:1000), p38 (Cat. No. 8690; dilution 1:1000), p-p38 (Thr180/Tyr182) (Cat. No. 4511; dilution 1:1000), and horseradish peroxidase (HRP)-linked secondary antibodies (Cat. No. 7074; dilution 1:2000) were purchased from Cell Signaling Technology (Danvers, MA, USA). Specific primary antibodies against SREBP-1c (Cat. No. PA1-337; dilution 1:1000) and LPL (Cat. No. PA5-85126; dilution 1:1000) were acquired from Invitrogen (Waltham, MA, USA).

### 2.2. Cell Culture and Adipocyte Differentiation

Human PCS-210-010 preadipocyte and mouse embryonic preadipocyte 3T3-L1 cells obtained from the American Type Culture Collection (ATCC; Manassas, VA, USA) were, respectively, cultured in FBM and DMEM containing 10% FBS, 100 units/mL of penicillin/streptomycin, and 2 mmol/L of l-glutamine under humidified conditions of 5% CO_2_ at 37 °C. For a differentiation program to convert preadipocytes to adipocytes, preadipocytes growing as monolayers up to 90% confluent for 2 days were exposed to a differentiation medium made of FBM or DMEM containing 10% FBS, 0.5 mM IBMX, 1 μM dexamethasone, and 5 μg/mL insulin for 2 days. At this stage, various concentrations of isopanduratin A were added, while 0.5% (*v*/*v*) DMSO was used as vehicle control. The differentiation medium was replaced with culture medium supplemented with 5 μg/mL of insulin. After further incubation for 2 days, cells were maintained in complete medium, which was changed every 2 days until lipid-droplet-containing adipocytes were observed under the microscope. Undifferentiated and differentiated cells were defined as negative control and positive control groups, respectively.

### 2.3. Cytotoxicity Assay

Following the recommended course of action, the cytotoxicity of isopanduratin A was evaluated using a crystal violet colorimetric assay [33]. Cells were seeded in a 96-well plate at a density of 1 × 10^4^ cells/well and incubated under humidified 5% CO_2_ at 37 °C overnight and then exposed for 48 h to isopanduratin A in a range of final concentrations (0–100 μM). A vehicle control (0.5% (*v*/*v*) DMSO) was also included. Dead detached cells were removed after washing twice with phosphate buffer saline (PBS; pH 7.4). The adherently viable cells were then stained with crystal violet solution (0.05% *w*/*v*) for 30 min at room temperature after being fixed with 10% *w*/*v* formic aldehyde for 30 min. The assayed plate was washed twice with deionized water to remove any excess crystal violet solution and then left to dry overnight. The stained cells were treated with 100 μL of methanol prior to absorbance measurement (570 nm) with a microplate reader (Anthros, Durham, NC, USA). The percentage of cell viability was calculated using the absorbance value of each treatment relative to that of the vehicle control.

### 2.4. Cell Proliferation Assay and Cell Cycle Analysis

The ability of 3T3-L1 cells to proliferate in the presence of isopanduratin A at its non-cytotoxic doses for 24–72 h was investigated by crystal violet staining [33,36]. 3T3-L1 cells (3.5 × 10^3^ cells/well in a 96-well plate) growing as a monolayer for 2 days were exposed to differentiation medium containing varying concentrations of isopanduratin A (0–10 μM) and incubated for 24, 48, and 72 h. A vehicle (0.5% (*v*/*v*) DMSO) was also included. At the end of each incubation period, the crystal violet staining assay was carried out as described previously, and the ability of cells to proliferate was calculated and reported as the percentage of cell proliferation in each treatment relative to that of the vehicle control measured at 24 h.

The impact of isopanduratin A on the passage of 3T3-L1 cells through the cell cycle was analyzed by flow cytometry. Cells seeded in a 6-well plate and at 90% confluent of their growth were treated with non-cytotoxic doses of isopanduratin A for 18 h. Undifferentiated or differentiated control cells were established by exposure to 0.5% (*v*/*v*) DMSO. Cells in each treatment and control were harvested by centrifugation for 5 min at 2500× *g* and 4 °C and then fixed overnight in 1 mL of ice-cold 70% (*v*/*v*) ethanol at −20 °C. The fixed cells were washed with PBS (pH 7.4), stained with 50 μg/mL PI solution (400 μL) containing 5 μg/mL DNase-free RNase solution for 30 min at room temperature, and kept away from light. DNA content was analyzed by flow cytometry (EMD Millipore, Austin, TX, USA). The percentages of cells in the G0/G1, S, and G2/M phases were then calculated using the FlowJo V10 software trial version (Williamson Way, Ashland, OR, USA).

### 2.5. Assessment of Cellular Lipid Content

The impact of isopanduratin A, at varying non-toxic doses, on the formation of lipid droplets in 3T3-L1 and PCS-210-010 adipocytes was evaluated by the Oil Red O staining assay. Both adipocytic cells undergoing the differentiation program, as described previously, were fixed with 10% formaldehyde for 30 min at room temperature, and then the fixed cells were stained with Oil Red O solution (at an Oil Red O:distilled water ratio of 6:4) for 1 h at room temperature. The stained cells were washed twice with 60% (*v*/*v*) isopropanol and randomly photographed under an inverted light microscope (Nikon Ts2, Tokyo, Japan). Intracellular Oil Red O-stained lipid droplets were eluted using 100% isopropanol, and their absorbance values at 500 nm wavelength were measured using a microplate reader (Anthros, Durham, NC, USA).

The effects of isopanduratin A at varying non-cytotoxic doses on cellular triglyceride and released glycerol levels were also determined, respectively, using triglyceride and glycerol assay kits (Sigma Aldrich, St. Louis, MO, USA), in accordance with the instructions of the manufacturer. Undifferentiated or differentiated cells treated with DMSO (0.5% *v*/*v*) functioned as controls for each experiment.

### 2.6. Western Blotting

The effects of isopanduratin A (0–10 μM) on the expression of proteins related to adipogenesis after 48 h of incubation were tracked by western blot analysis. Undifferentiated and differentiated 3T3-L1 cells treated with DMSO (0.5% *v*/*v*) functioned as controls. Cells were collected and lysed on ice in RIPA buffer supplemented with a protease inhibitor cocktail for 45 min. Cell lysates were quantified for protein concentration using the BCA assay and stored at −80 °C until further use. Equal protein samples (30 μg) were loaded to separate on 10% SDS-PAGE and transferred onto a nitrocellulose membrane (BIO-RAD, Hercules, CA, USA). The membranes were blocked in 5% skim milk for 1 h at room temperature and incubated overnight with primary antibodies at 4 °C. The membranes were then washed (7 min × 3 times) with Tris-buffered saline with 0.1% Tween^®^ 20 (TBST) before incubation with HRP-conjugated secondary antibody for 2 h at room temperature. The membranes were washed 3 times with TBST to remove excess antibodies and detected using western chemiluminescent ECL substrates. The protein expression level was calculated as the ratio of the band intensity of the target protein to that of β-actin—a housekeeping protein.

### 2.7. Reverse Transcription-Quantitative Polymerase Chain Reaction (RT-qPCR)

The impact of isopanduratin A on the expression of some proteins involved in the differentiation of 3T3-L1 adipocytes was confirmed at the transcriptional level using the RT-qPCR technique. 3T3-L1 preadipocytes (5 × 10^4^ cells/well in a 6-well plate) with up to 90% confluent were treated with varying non-cytotoxic doses of isopanduratin A for 2 days in differentiation medium. Undifferentiated and differentiated 3T3-L1 cells treated with DMSO (0.5% (*v*/*v*) functioned as controls for this study. The medium was removed, and the cells were rinsed thrice with ice-cold PBS (pH 7.4) and extracted for their RNA using the PureLink^™^ RNA Mini Kit (Invitrogen, Carisbad, CA, USA). An equal amount (1 μg) of total RNA was reverse-transcribed to complementary DNA with a RevertAid first-strand cDNA synthesis kit (Thermo Scientific Pierce, Rockford, IL, USA). The Bio-Rad Luna Universal qPCR master mix (Hercules, CA, USA) was used in the assay reaction, while amplification was performed with the Bio-Rad CFX96 Touch real-time PCR detection system (Hercules, CA, USA), in accordance with the instructions of the manufacturer. The RT-qPCR primers (Table 1) and conditions were previously described elsewhere [36]. The expression level of each target gene was normalized with that of *Gapdh*—a housekeeping gene. Relative mRNA expression levels were analyzed using the 2^−(ave.∆∆*C*^_T_^)^ method, where *C*_T_ is the threshold cycle.

### 2.8. Statistical Analysis

All experiments were carried out in triplicate, and the results are expressed as mean ± standard deviation (SD). Statistical comparison of means by one-way analysis of variance (ANOVA) with Tukey’s post hoc test was performed using GraphPad Prism 8.0.2 software (San Diego, CA, USA). A *p*-value of <0.05 was considered statistically significant.

## 3. Results

### 3.1. Effect of Isopanduratin A on Adipogenesis in 3T3-L1 Preadipocytes

In this study, murine 3T3-L1 preadipocyte cells, which can differentiate into mature adipocytes under appropriate conditions [4,37], were used. Initially, the toxicity of each test compound was evaluated at 5 μM by a crystal violet assay, as previously described [33]. At this concentration, pinostrobin (**1**), panduratin A (**3**), isopanduratin A (**4**), and cardamonin (**6**) were all non-toxic and showed a significant reduction in intracellular lipid content in the Oil Red O staining assay (Table 2), suggesting their anti-adipogenic potential. Isopanduratin A showed a drop in the percentage of stained cells to approximately 60%, compared to the vehicle control. The cytotoxic effect of isopanduratin A was then further assessed in a wider range of concentrations (0–100 μM). The highest non-toxic dose was found to be 10 μM, and the half-maximum inhibitory concentration was 28.63 ± 0.70 μM.

**Figure 1:**
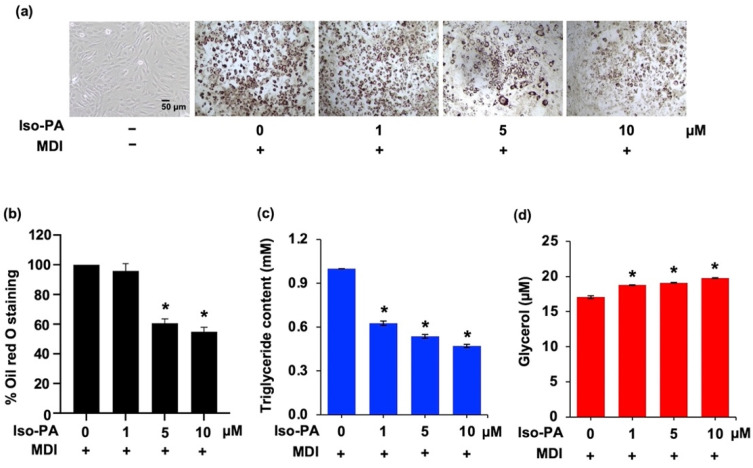
Inhibitory effects of isopanduratin A on the accumulation of intracellular lipids in differentiated adipocytes. Mouse preadipocyte 3T3-L1 cells were cultured in differentiation medium in the presence or absence of isopanduratin A at non-toxic concentrations (1–10 μM). (**a**) The lower number of cellular lipid droplets stained with Oil Red O and (**b**) the relative absorbance of the eluted Oil Red O dye at 500 nm were noticed in differentiated 3T3-L1 cells treated with 5–10 μM isopanduratin A for 48 h. The altered levels of (**c**) intracellular triglyceride content and (**d**) extracellular glycerol were initially detected in differentiated 3T3-L1 cells treated with 1 μM isopanduratin A. Confluent 3T3-L1 cells were cultured in the presence (+) or absence (−) of differentiation medium (MDI) with 0–10 μM isopanduratin A (Iso-PA). Cells treated with dimethyl sulfoxide (0.5% *v*/*v*) functioned as the untreated control (0). Bar graphs demonstrating mean ± SD (n = 3) were created using GraphPad Prism. A one-way ANOVA with Tukey’s post hoc test was used to compare the means of treatment and differentiated control groups (* *p <* 0.05).

The dose-dependent effect of isopanduratin A on 3T3-L1 adipocyte differentiation was then further examined by measuring the accumulation of cellular lipid droplets stained with Oil Red O dye (Figure 1a). Figure 1b shows that isopanduratin A at 5 and 10 μM inhibited cell differentiation in a dose-dependent manner, as indicated by the lower percentage of stained lipid droplets. The intracellular triglyceride content in the cells exposed to 1–10 μM isopanduratin A for 48 h decreased significantly, compared to untreated control cells (Figure 1c), although a reduction in cellular lipid droplets by 1 μM isopanduratin A was not clearly observed. Similarly, isopanduratin A at 1–10 μM significantly increased the amount of extracellular glycerol released from differentiated cells (Figure 1d).

**Figure 2:**
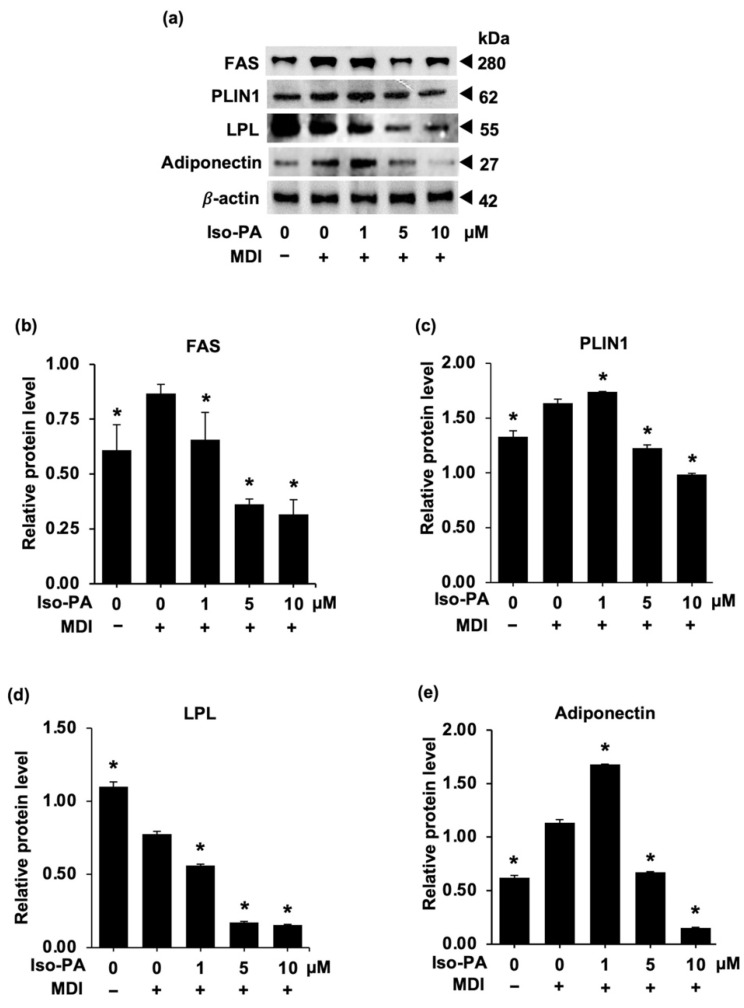
Inhibitory effects of isopanduratin A on the expression of adipogenic effectors in 3T3-L1 adipocytes. Cells were differentiated for 8 days in differentiation medium containing isopanduratin A at non-toxic concentrations. (**a**) Protein expression was then examined by Western blotting. The expression levels of (**b**) FAS, (**c**) PLIN1, (**d**) LPL, and (**e**) adiponectin relative to β-actin were measured by the ImageJ program. Confluent 3T3-L1 cells were cultured in the presence (+) or absence (−) of differentiation medium (MDI) with 0–10 μM isopanduratin A (Iso-PA). Cells treated with 0.5% *v*/*v* DMSO functioned as untreated control (0). Bar graphs demonstrating mean ± SD (n = 3) were created using GraphPad Prism. A one-way ANOVA with Tukey’s post hoc test was used to compare the means of treatment and the differentiated control groups (* *p <* 0.05).

The expression of proteins related to lipid metabolism as markers of mature adipocytes was further investigated in differentiated cells. Elevated expression levels of FAS, LPL, PLIN, and adiponectin, which play an important role in lipogenesis, were clearly observed in cells cultured with differentiation medium for 8 days (Figure 2a). Intriguingly, 5–10 μM of isopanduratin A significantly suppressed the expression of PLIN (Figure 2c) and adiponectin (Figure 2e) in differentiated cells, while lower levels of FAS (Figure 2b) and LPL (Figure 2d) were observed at as low as 1 μM of isopanduratin A. These results demonstrated that isopanduratin A at non-cytotoxic doses could efficiently limit lipogenesis during cell differentiation.

### 3.2. Isopanduratin A Inhibits Mitotic Clonal Expansion during Adipogenesis

Preadipocytes undergo mitotic clonal expansion (MCE) during the early stage of adipogenesis. Before the beginning of cell differentiation, these growth-arrested preadipocytes usually undergo a few rounds of mitosis. Concurrent reentry into the cell cycle caused by MCE leads to an increased number of adipocytes [7]. MCE is mediated by the activation of cyclin-dependent kinase (CDK) and cyclin family proteins. Following MCE, activated C/EBPβ stimulates C/EBPα, which in turn causes PPARγ to begin transcription [36,38].

**Figure 3:**
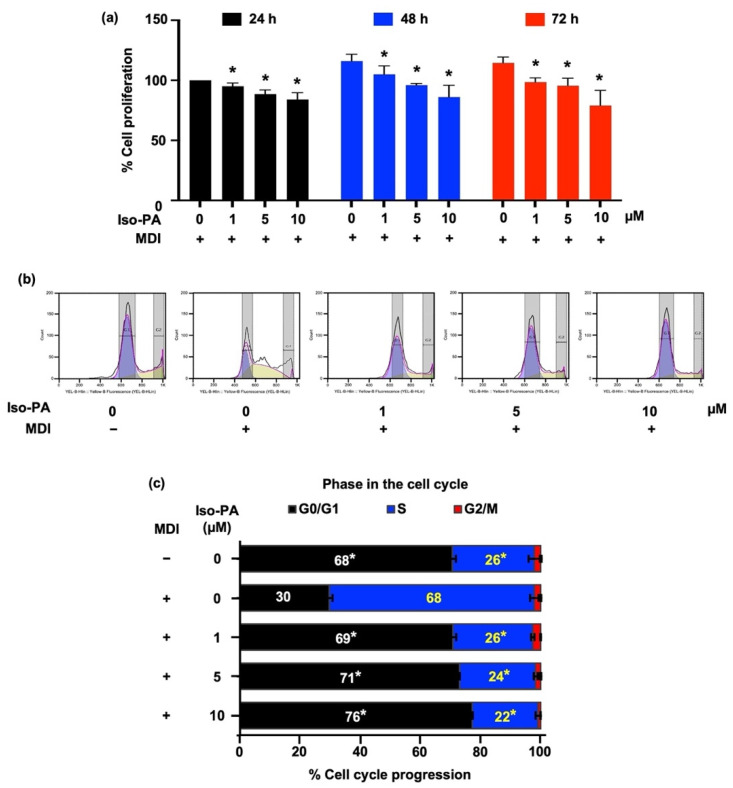
Effects of isopanduratin A on cell proliferation and cell cycle progression of differentiated 3T3-L1 cells. (**a**) A crystal violet assay was used to track the proliferation of cells cultured in differentiation medium with or without 1–10 μM isopanduratin A for 24 to 72 h. The alteration of the cell cycle in cells treated with isopanduratin A was evaluated by flow cytometry and presented in (**b**) histograms and (**c**) cell frequency in each phase. Confluent 3T3-L1 cells were cultured in the presence (+) or absence (−) of differentiation medium (MDI) with 0–10 μM isopanduratin A (Iso-PA). Cells treated with dimethyl sulfoxide (0.5% *v*/*v*) functioned as the untreated control (0). Bar graphs demonstrating mean ± SD (n = 3) were created using GraphPad Prism. A one-way ANOVA with Tukey’s post hoc test was used to compare the means of treatment and the differentiated control groups (* *p <* 0.05).

As presented in Figure 3a (see Figure S1), isopanduratin A (1–10 μM) significantly inhibited the proliferation of 3T3-L1 preadipocytes after incubation for 24, 48, and 72 h, compared to differentiated control cells at each time point. The effect of isopanduratin A on cell cycle progression during MCE was further determined. The number of cells at different stages of the cell cycle was assessed after culture in differentiation medium for 18 h in the presence or absence of 1–10 μM of isopanduratin A. The histograms obtained from flow cytometry reveal the entry into the S phase of the cell cycle in differentiated 3T3-L1 cells (Figure 3b). Surprisingly, isopanduratin A significantly hindered the progression of the cell cycle, as indicated by the higher number of cells in the G0/G1 phase, compared to the differentiated control group (Figure 3c).

**Figure 4:**
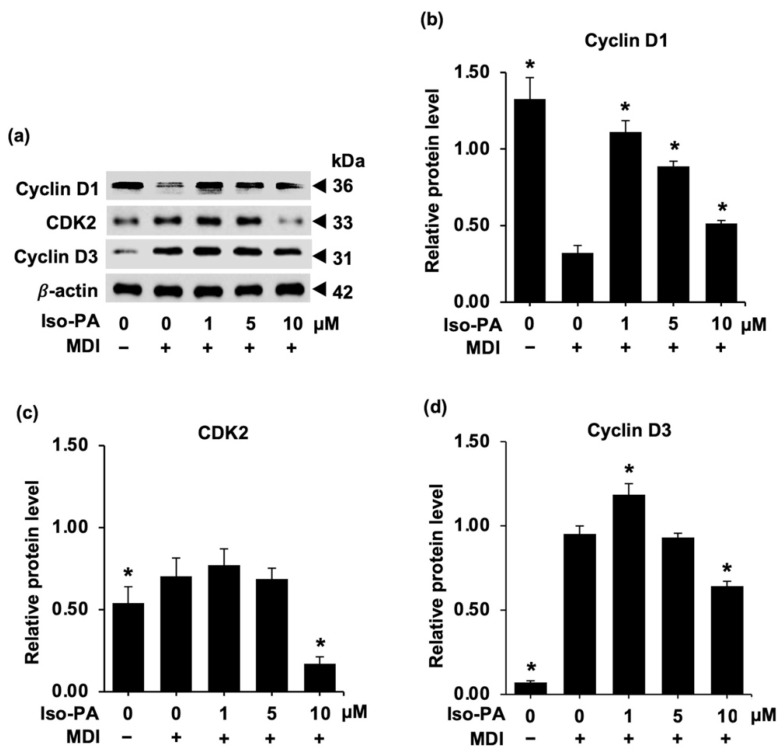
Isopanduratin A alters cell-cycle-regulating proteins during differentiation of 3T3-L1 adipocytes. The band intensity of the target protein obtained from (**a**) Western blot analysis was estimated by comparison with that of β-actin (internal reference) including (**b**) cyclin D1, (**c**) CDK 2, and (**d**) cyclin D3. Confluent 3T3-L1 cells were cultured in the presence (+) or absence (−) of differentiation medium (MDI) with 0–10 μM isopanduratin A (Iso-PA). Cells treated with 0.5% *v*/*v* DMSO functioned as untreated control (0). Bar graphs demonstrating mean ± SD (n = 3) were created using GraphPad Prism. A one-way ANOVA with Tukey’s post hoc test was used to compare the means of treatment and differentiated control groups (* *p <* 0.05).

Figure 4 shows that isopanduratin A markedly altered the expression of MCE-mediated proteins (cyclins D1 and D3 and CDK2) in differentiated 3T3-L1 cells after 18 h of incubation, as proven by western blot analysis. Cyclin D1 is known to be suppressed, while other cyclin proteins are upregulated, during the initial phase of adipogenesis [39]. Cyclin D1 inhibits adipogenesis by preventing the expression of C/EBPα [40]. In this study, a reduction in cyclin D1 levels was observed in differentiated 3T3-L1 cells, but this downregulation was effectively reversed by isopanduratin A (Figure 4a,b) (See Figure S2). Lower levels of CDK2 (Figure 4c) and cyclin D3 (Figure 4d) were found in cells treated with isopanduratin A (10 μM) in comparison with the differentiated control group. These observations indicate that isopanduratin A delayed cell passage in the cell cycle by modulating MCE-mediated protein expression.

### 3.3. Isopanduratin A Downregulates Adipogenic Transcription Factors

**Figure 5:**
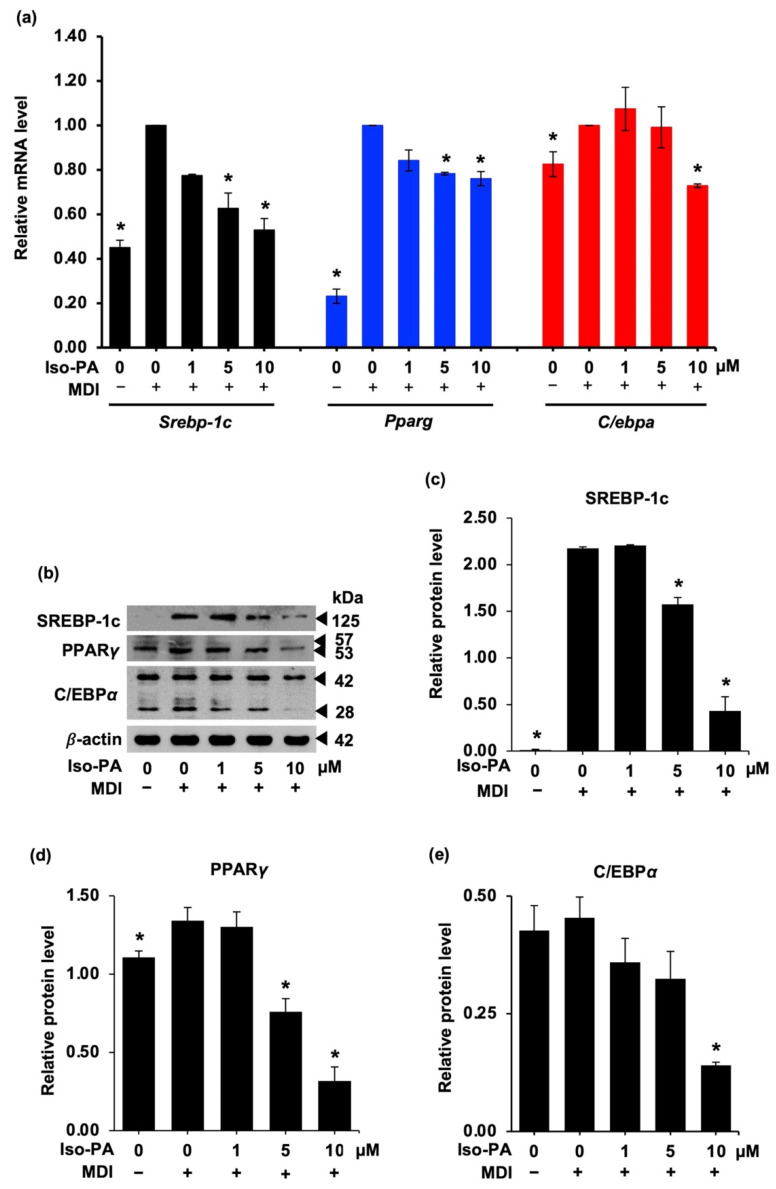
Isopanduratin A downregulates adipogenic transcription factors in differentiated 3T3-L1 cells. (**a**) Relative mRNA levels analyzed via RT-qPCR demonstrated the gene expression of *C*/*ebpa*, *Pparg*, and *Srebp-1c*. (**b**) Western blotting was used to evaluate the protein expression level. Treatment with isopanduratin A significantly decreased the levels of (**c**) SREBP-1c, (**d**) PPARγ, and (**e**) C/EBPα proteins in differentiated 3T3-L1 cells. Confluent 3T3-L1 cells were cultured in the presence (+) or absence (−) of differentiation medium (MDI) with 0–10 μM isopanduratin A (Iso-PA). Cells treated with 0.5% *v*/*v* DMSO functioned as untreated control (0). Bar graphs demonstrating mean ± SD (n = 3) were created using GraphPad Prism. A one-way ANOVA with Tukey’s post hoc test was used to compare the means of treatment and differentiated control groups (* *p <* 0.05).

To further elucidate the molecular mechanisms underlying the suppressive effect of isopanduratin A on adipogenesis, the expression of various adipogenic transcription factors was determined at both the mRNA and protein expression levels. Preadipocyte 3T3-L1 cells were collected during the early differentiation stage after 48 h of incubation with or without differentiation medium with isopanduratin A at non-toxic concentrations. Upregulated levels of transcription factor mRNA, including PPARγ, SREBP-1C, and C/EBPα, were observed in cells cultured in differentiation medium for 48 h (Figure 5a) (see Figure S3). Nevertheless, isopanduratin A at 5 and 10 μM significantly decreased the levels of SREBP-1C and PPARγ mRNA, compared to those of the differentiated control cells. It should be noted that the decreased level of C/EBPα mRNA was observed only in the 3T3-L1 cells incubated with isopanduratin A at a high concentration (10 μM). Consistent with the mRNA levels detected by qRT-PCR, western blotting revealed lower expression levels of the SREBP-1C, PPARγ, and C/EBPα proteins in the differentiated 3T3-L1 cells cultured with 5–10 μM of isopanduratin A, compared to differentiated control groups (Figure 5b–d).

### 3.4. Upstream Signals from MAPKs Are Modulated by Isopanduratin A

Mitogen-activated protein kinases (MAPKs), including ERK, p38, and JNK, play an important role during adipogenesis, in which their regulating roles, such as cell proliferation and differentiation, are exerted [37]. Suppression of MAPK signaling molecules efficiently inhibits adipocyte development, and it has been demonstrated that altering these biomolecules during adipocyte differentiation is one of the promising strategies to slow adipogenesis and cellular lipid metabolism [16].

**Figure 6:**
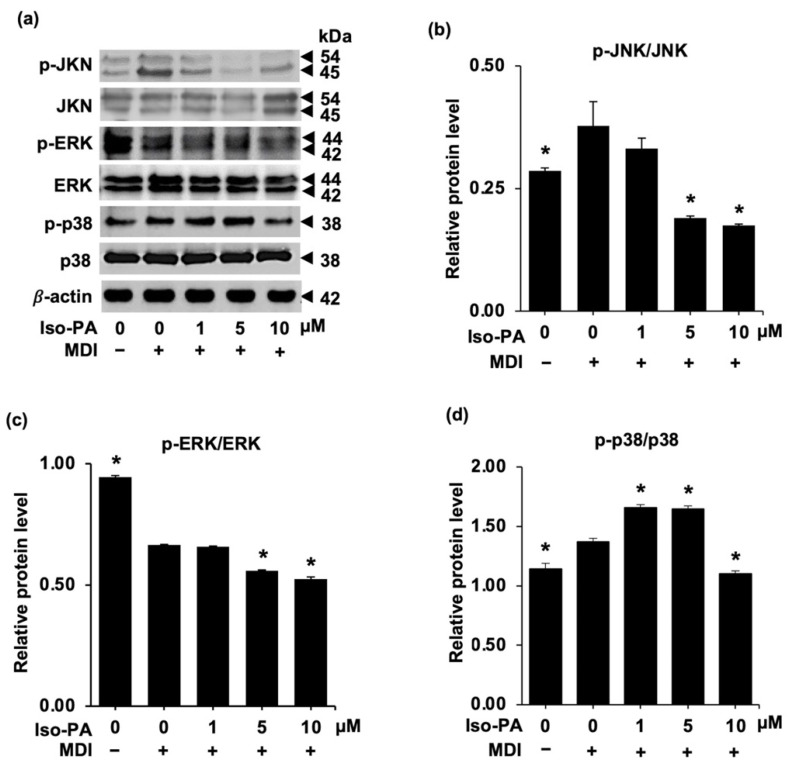
Isopanduratin A deactivates MAPK signaling molecules in differentiated 3T3-L1 cells. (**a**) After 48 h of incubation in differentiation medium containing 1–10 μM isopanduratin A, cells were subjected to Western blot analysis. The phosphorylation ratios of (**b**) p-JNK/JNK, (**c**) p-ERK/ERK, and (**d**) p-p38/p38 were significantly lower in cells treated with isopanduratin A, compared to the differentiated control groups. Confluent 3T3-L1 cells were cultured in the presence (+) or absence (−) of differentiation medium (MDI) with 0–10 μM isopanduratin A (Iso-PA). Cells treated with 0.5% *v*/*v* DMSO functioned as the untreated control (0). Bar graphs demonstrating mean ± SD (n = 3) were created using GraphPad Prism. A one-way ANOVA with Tukey’s post hoc test was used to compare the means of treatment and differentiated control groups (* *p <* 0.05).

In the present investigation, a western blot analysis was performed to determine whether isopanduratin A modulates the signaling molecules in the MAPK pathway (Figure 6a). The decreased levels of p-JNK/JNK (Figure 6b) (see Figure S4) and p-ERK/ERK (Figure 6c) were clearly indicated in the 3T3-L1 cells cultured with differentiation medium containing 5–10 μM of isopanduratin A, compared to differentiated control cells. It is worth noting that isopanduratin A at a high concentration (10 μM) dramatically suppressed p-p38/p38 signaling (Figure 6d). Thus, these results indicated that isopanduratin A might attenuate adipogenesis by inhibiting the MAPK pathway.

### 3.5. Isopanduratin A Modulates the Crosstalk between AMPK-ACC and AKT/GSK3β Signals

**Figure 7:**
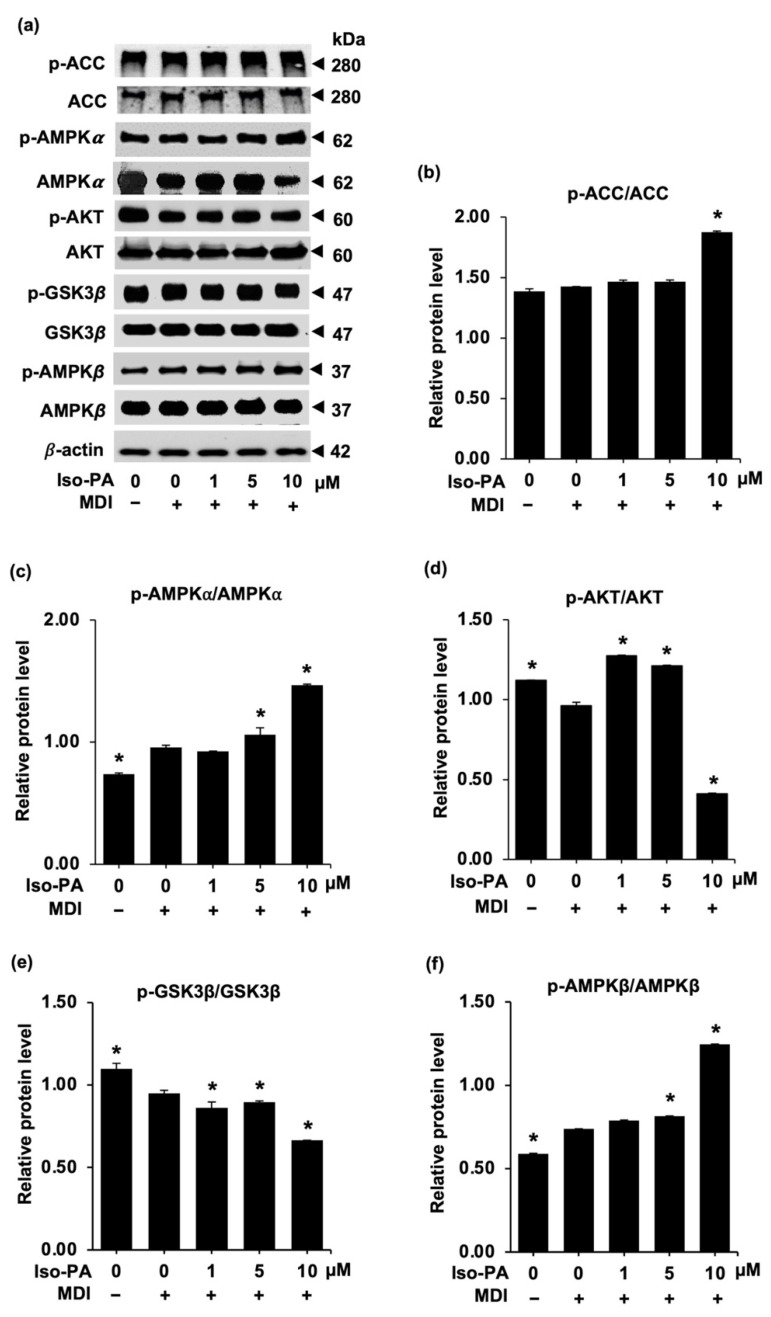
Regulatory effects of isopanduratin A on AKT-related signaling pathways. Isopanduratin A activates the AMPK-ACC pathway but deactivates the AKT/GSK3β signaling pathway in differentiating 3T3-L1 cells. The band intensity of each protein obtained from (**a**) Western blotting was used to analyze the ratio of the phosphorylated to the unphosphorylated form of (**b**) p-ACC/ACC (**c**) p-AMPKα/AMPKα, (**d**) pAKT/AKT, (**e**) p-GSK3β/GSK3β, and (**f**) p-AMPK β/AMPK β. Confluent 3T3-L1 cells were cultured in the presence (+) or absence (−) of differentiation medium (MDI) with 0–10 μM isopanduratin A (Iso-PA). Cells treated with 0.5% *v*/*v* dimethyl sulfoxide served as the untreated control (0). Bar graphs demonstrating mean ± SD (n = 3) were created using GraphPad Prism. A one-way ANOVA with Tukey’s post hoc test was used to compare the means of treatment and differentiated control groups (* *p <* 0.05).

Several reports suggest that AMP-activated protein kinase (AMPK) regulates the cellular energy balance by inhibiting lipogenesis and promoting lipolysis [41,42]. In this study, isopanduratin A affected AMPK signaling molecules, as illustrated by Western blot analysis (Figure 7a) (see Figures S5 and S6). The activation of the AMPK pathway by this compound was indicated by the highly elevated levels of p-ACC/ACC (Figure 7b), p-AMPKα/AMPKα (Figure 7c), and p-AMPKβ/AMPKβ (Figure 7f) in the differentiated 3T3-L1 cells cultured with 10 μM of isopanduratin A for 48 h.

Protein kinase B (AKT) is another upstream molecule that plays an important role in adipogenesis. Phosphorylated AKT (p-AKT) suppresses AMPK-ACC signals, resulting in the upregulation of adipogenic transcription factors and promotion of lipogenesis [43]. Additionally, p-GSK3β, which mediates the transcription of adipogenic transcription factors, is also modulated by p-AKT. The AKT/GSK3β cascade is required for the expression of C/EBPβ, C/EBPα, and PPARγ during cell differentiation [44]. Consistent with the elevated expression of AMPK-ACC signals and decreased levels of adipogenic transcription factors, the ratios of p-AKT/AKT (Figure 7d) and p-GSK3β/GSK3β (Figure 7e) were suppressed by isopanduratin A. These results suggest that isopanduratin A modulates the signaling pathways of AKT/GSK3β and AKT/AMPK-ACC to inhibit adipogenesis.

### 3.6. Isopanduratin A Suppresses Adipocyte Maturation in Human Preadipocytes

**Figure 8:**
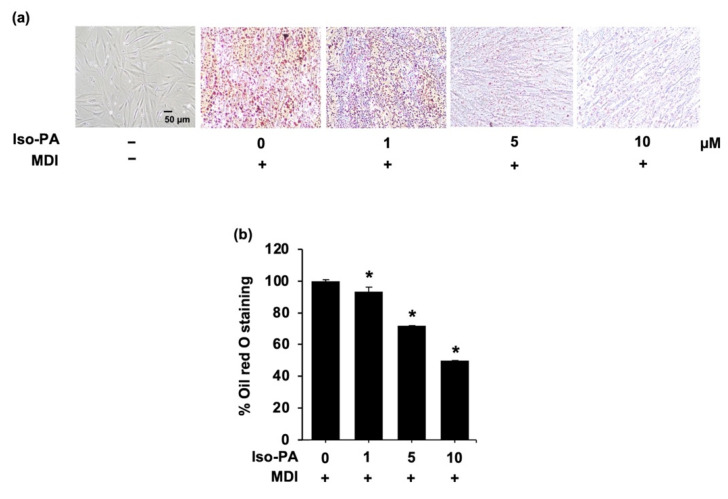
The suppressive effect of isopanduratin A on lipid accumulation in differentiated human PCS-210-010 preadipocytes cultured with isopanduratin A was assessed by Oil Red O staining and represented as percentage of Oil Red O staining. (**a**) Confluent 3T3-L1 cells were cultured in the presence (+) or absence (−) of differentiation medium (MDI) with 0–10 μM isopanduratin A (Iso-PA). Cells treated with 0.5% *v*/*v* DMSO functioned as untreated control (0). (**b**) Bar graphs demonstrating mean ± SD (n = 3) were created using GraphPad Prism. A one-way ANOVA with Tukey’s post hoc test was used to compare the means of treatment and differentiated control groups (* *p <* 0.05).

The antiadipogenic potential of isopanduratin A was further studied in primary human PCS-210-010 preadipocytes. The lipid contents were analyzed by Oil Red O staining (Figure 8a). Treatment with isopanduratin A at 1, 5, and 10 μM decreased the number of cellular lipid droplets by 93.51%, 71.75%, and 49.79%, respectively (Figure 8b). These results suggested that isopanduratin A suppresses adipogenesis in human preadipocytes in a dose-dependent manner.

## 4. Discussion

Obesity is associated with the onset of metabolic syndrome and various degenerative diseases that can cause various chronic health problems and often lead to premature death. During the recent COVID-19 pandemic, obesity increased the risk of hospitalization and admission to intensive care units [45]. The unusual expansion of adipose tissue, a characteristic feature of obesity, depends on adipocyte hypertrophy (an increase in cell size) and/or hyperplasia (an increase in cell number) [46]. It is commonly acknowledged that a long-term regulated lifestyle that involves reducing food intake and increasing physical activity can effectively lower body weight. However, these diet and lifestyle modifications are challenging for many overweight patients. Currently, nutrition intervention is highlighted as an alternative strategy to treat obesity [47].

In this study, in the Oil Red O staining assay, isopanduratin A at non-toxic concentrations reduced the number of mature, lipid-containing adipocytes in both mouse 3T3-L1 (Figure 1a,b) and human PCS-210-010 (Figure 8) preadipocyte models. These results indicate its anti-adipogenic activity. It should be noted that isopanduratin A at 1 μM could reduce cellular fat accumulation in human preadipocytes more than in murine preadipocytes. Lipid metabolism plays a crucial role in adipocyte differentiation, and its dysregulation is a critical factor in the development of obesity [48]. The decrease in intracellular triglyceride content and the elevated levels of released glycerol (Figure 1c,d) demonstrated the lipolytic effect of isopaduratin A.

The suppressive effect of isopanduratin A on 3T3-L1 adipogenesis is further evidenced by decreased expression levels of adipogenic effectors, including FAS, PLIN1, LPL, and adiponectin (Figure 2). These lipid-metabolism-modulating proteins are essential for maintaining cellular lipid homeostasis and are associated with various metabolic conditions such as hyperlipidemia, insulin resistance, atherosclerosis, and obesity [9,37,48,49,50,51,52]. Due to its ability to modulate cellular lipid accumulation and interact with these lipid metabolism proteins, isopanduratin A might be a potential nutraceutical candidate for the treatment of several metabolic diseases.

Mitotic clonal expansion (MCE) is the process in which the number of premature adipocytes increases as a result of cell cycle re-entry and the repeated cycles (two–three cycles) of cell proliferation at the early stage of adipogenesis [7]. Several natural compounds that possess an anti-adipogenic potential exhibit cell cycle arrest in differentiated preadipocytes [37,38,39]. As mentioned above, growth-arrested preadipocytes undergo MCE, which is mediated by the activation of cyclin/CDK complexes [7]. Interestingly, treatment with 1–10 μM of isopanduratin A showed a significant decrease in the percentage of cell proliferation, compared to the differentiation control cells (Figure 3a). Increased cyclin D1 expression in preadipocytes treated with isopanduratin A, with concomitant lowered levels of cyclin D3 and CDK2 (Figure 4), indicated cell cycle arrest in the G0/G1 phase. Similar effects on cyclin D1 levels were reported earlier for other natural polyphenols such as delphinidin and curcumin, both of which are strong anti-adipogenic compounds [53,54]. The increase in cyclin D1 levels may suggest that isopanduratin A also inhibits adipogenesis by activating the Wnt/β-catenin signaling pathway. Consistent with the change in the DNA content analyzed by flow cytometry, the accumulation of G0/G1 cells and the decrease in S phase cells occurred in differentiated preadipocytes cultured with isopanduratin A at 1–10 μM (Figure 3b,c). These results suggested that isopanduratin A inhibited the generation of mature adipocytes from preadipocytes by triggering cell cycle arrest.

After the MCE period, activation of C/EBPα triggers PPARγ transcription in association with the expression of adipogenesis-regulating proteins [36]. During adipocyte differentiation, transcription factors C/EBPα, PPARγ, and SREBP-1c cross-activate one another to exert their adipogenic functions [38,55]. Previous studies showed that C/EBPα controls the expression of SREBP-1c and that low C/EBPα levels lead to reduced PPARγ activity. In addition, gene expressions related to cellular lipid storage and insulin response are affected by C/EBPα [56,57]. Intriguingly, isopanduratin A suppressed adipogenesis in 3T3-L1 cells by downregulating these transcription factors at both the translation and transcription levels (Figure 5).

The expression of adipogenic transcription factors is also governed by the opposite correlation between the AKT/GSK3β and the AMPK-ACC pathways. As these two pathways critically mediate the upstream machinery of adipocyte differentiation [58], the regulation of proteins involved in these processes could be another mechanism for suppressing adipogenesis. It is plausible that AMPK and AKT are competitively phosphorylated by an energy balance sensor that controls several metabolic pathways [59]. The AKT/GSK3β cascade is vital for the expressions of C/EBPβ, C/EBPα, and PPARγ during cell differentiation [60].

Moreover, the AMPK pathway influences the expression of FAS and FABP4, which participate in lipogenesis at the late stage of adipogenesis [57]. In mouse and human mesenchymal cells, upregulated levels of C/EBPα, PPARγ, and SREBP-1c are caused by the downregulation of AMPK, which also affects the activation of ACC [55]. Activation of AMPK (p-AMPK), in association with ACC initiation, hampers triglyceride and fatty acid production by suppressing SREBP-1c and FAS during adipogenesis [47,59]. Therefore, the good correlation between the suppressive effects of isopanduratin A on adipogenic proteins (Figure 4 and Figure 5) and the downregulated levels of p-AKT and p-GSK3β as well as the upregulated levels of p-AMPK and p-ACC (Figure 7) suggests that the compound inhibits adipogenesis and lipogenesis in mature adipocytes through the AKT/AMPK-ACC pathway.

In general, extracellular stimuli can induce MAPK signaling, which, in turn, activates several intracellular responses through the phosphorylation of specific sites and components, including ERK, JNK, and p38. Studies showed that adipogenic transcription regulators are influenced by proteins in the MAPK family [61]. In this study, isopanduratin A decreased the phosphorylated forms of JNK, ERK, and p38 (Figure 6). Interestingly, isopanduratin A suppressed MAPK signaling concomitantly with a reduction in intracellular lipid accumulation (Figure 1). ERK phosphorylation is known to be essential for cell proliferation and cell cycle progression during the MCE process [62,63,64]. Isopanduratin A prevented MCE, in parallel with the downregulated levels of p-ERK/ERK (Figure 6c). On the other hand, in our previous report, pinostrobin did not suppress MCE, in agreement with its lack of activity on p-ERK/ERK [33]. Panduratin A and cardamonin, the other adipogenic suppressors obtained from fingerroot, have never been reported for MCE interference.

It is worth noting that the non-theoretical alteration of the upstream regulating molecules (p-AKT, p-GSK3β, p-AMPK, p-ACC, and p-ERK) observed in this study could be the result of late-stage detection. However, isopanduratin A indeed restricts these signaling pathways during adipogenesis. Although more in-depth investigations are needed, the overall results suggest that isopanduratin A suppresses adipogenesis through multi-target mechanisms.

## 5. Conclusions

**Figure 9:**
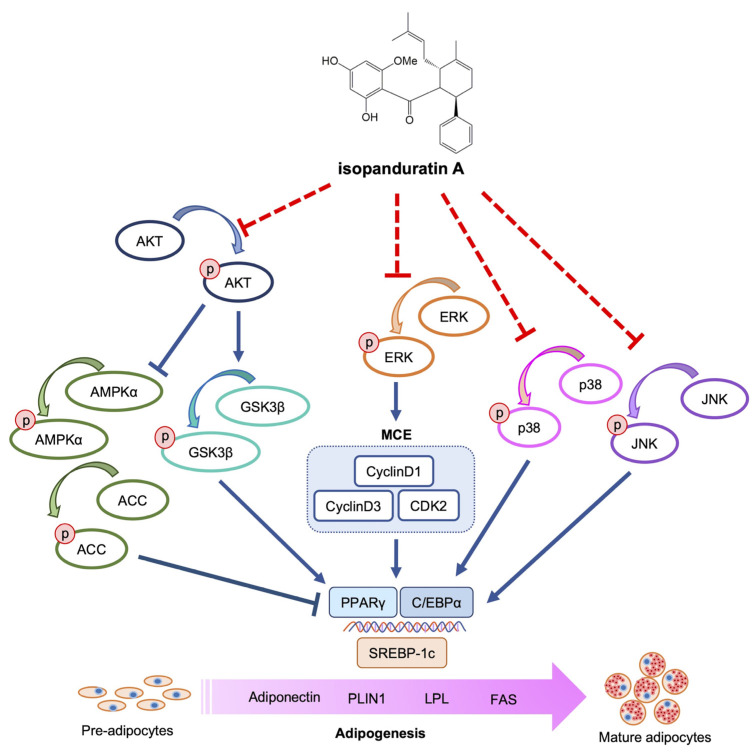
Proposed regulatory mechanisms of isopanduratin A on the suppression of adipogenesis. Isopanduratin A decreases adipocyte generation and cellular lipid accumulation by downregulating adipogenic effectors (FAS, PLIN1, LPL, and adiponectin) and adipogenic transcription factors (PPARγ, C/EBPα, and SREBP-1c). The multi-target inhibitory properties of isopanduratin A are evidenced by its modulation on mitotic clonal expansion-regulating proteins (CyclinD1, CyclinD3, and CDK2) and AKT (AKT/GSK3β and AKT/AMPK-ACC) and MAPK (JNK, ERK, and p38) signals.

Fingerroot (*Bosenbergia rotunda*) possesses pinostrobin, panduratin A, cardamonin, and idopanduratin A as adipogenic inhibitors. Isopanduratin A suppresses adipogenesis by modulating AKT/AMPK-ACC (AKT/GSK3β and AKT/AMPK-ACC) and MAPK (JNK/ERK/p38) signals that correspond to the downregulation of key adipogenic regulators (SREBP-1c, PPARγ, and C/EBPα) and adipogenic effectors (FAS, PLIN1, LPL, and adiponectin) (Figure 9). It is worth noting that isopanduratin A also inhibits MCE by preventing ERK phosphorylation at the early stage of adipogenesis. This property is absent in pinostrobin and has not yet been described for panduratin A or cardamonin. Taken together, our results shed light on the molecular mechanisms underlying the anti-adipogenic activity of isopanduratin A and provide further evidence for the potential use of fingerroot as a functional food against weight gain and obesity. Rigorous preclinical and clinical trials should be performed to establish this hypothesis. As a culinary plant, fingerroot might be consumed directly as a functional food or used as an ingredient in nutraceutical products for body weight control. However, the safety for long-term daily consumption, as well as the stability and bioavailability of the active principles, must be thoroughly investigated before any application can be realized.

## Figures and Tables

**Table 1:** RT-qPCR primers used in this study.

Targeted Gene	Primer	Nucleotide Sequence (5′-3′)
*Pparg*	PpargF	GATTCTCCTRTTGACCCAG
	PpargR	GAR TGSGAGTGGTCTTCCAT
*C*/*ebpa*	CebpaF	AGTCGGTGGACAAGAACAGC
	CebpaR	GTGTCCAGTTCRCGGCTCA
*Srebp1c*	Srebp1cF	YTGCMGACCCTGGTGAGTG
	Srebp1cR	GASCGGTAGCGCTTCTCAAT
*Gadph*	GADPHF	ACTCCACTCACGGCAAATTC
	GADPHR	TCTCCATGGTGGTGAAGACA

**Table 2:** List of phytochemicals from *Boesenbergia rotunda* roots and their effects on lipid content in 3T3-L1 cells determined by Oil Red O staining.

Tested Chemical *^a^*	Relative Percentage of Oil Red O Stained Cells (%) *^b^*
Vehicle control *^c^*	100.00 ± 0.00
(**1**) Pinostrobin [C_16_H_14_O_4_]	66.79 ± 2.34 *
(**2**) Geraniol [C_10_H_18_O]	106.46 ± 3.34
(**3**) Panduratin A [C_26_H_30_O_4_]	76.94 ± 1.14 *
(**4**) Isopanduratin A [C_26_H_30_O_4_]	64.04 ± 3.70 *
(**5**) Pinocembrin [C_15_H_12_O_4_]	117.31 ± 7.05
(**6**) Cardamonin [C_16_H_14_O_4_]	80.89 ± 5.58 *
(**7**) Hydroxypanduratin A [C_25_H_28_O_4_]	99.53 ± 0.59
(**8**) 5,6-Dehydrokawain [C_14_H_13_O_3_]	115.57 ± 2.89
(**9**) Rotundaflavanochalcone [C_31_H_26_O_8_]	115.05 ± 4.31
(**10**) 2′,4′,6′-Trihydroxydihydrochalcone [C_15_H_15_O_4_]	99.95 ± 3.92
(**11**) Alpinetin [C_16_H_15_O_4_]	108.18 ± 2.28
(**12**) Iso-rotundaflavanochalcone [C_31_H_26_O_8_]	86.31 ± 7.20

*^a^* All tested compounds (5 µM) with a purity of >98% were isolated from *B*. *rotunda* and identified by nuclear magnetic resonance spectroscopy and mass spectrometry, as previously described [31]. *^b^* The Oil Red O staining assay was conducted and reported as the percentage of Oil Red O-stained cells compared to that of the control. *^c^* Dimethyl sulfoxide at 0.5% (*v*/*v*) was used as a vehicle control. Each experiment was carried out in at least triplicate, and a one-way analysis of the variant-based comparison of means ± SD was carried out. An asterisk refers to the significant difference in means compared to control cells at *p* < 0.05.

## Data Availability

Data are contained within the article and Supplementary Material.

## Supplementary Materials

The following supporting information can be downloaded at https://www.mdpi.com/article/10.3390/foods12051014/s1, Figure S1: Original western blot images for Figure 3a; Figure S2: Original western blot images for Figure 4a; Figure S3: Original western blot images for Figure 5a; Figure S4: Original western blot images for Figure 6a; Figure S5: Original western blot images for Figure 7a; Figure S6: Original western blot images for Figure 7a (continued).
