# Supplementary material for: Inhibitory Effect of Isopanduratin A on Adipogenesis: A Study of Possible Mechanisms
Source: Foods. 2023 Feb 27;12(5):1014. doi: 10.3390/foods12051014 (PMC10000982; doi:10.3390/foods12051014)
Supplement: Supplementary file 1 [file foods-12-01014-s001.zip › foods-2216519-supplementary.pdf]

Supplementary Materials

Inhibitory Effect of Isopanduratin A on Adipogenesis: A study of Possible Mechanisms

Prapenpuksiri Rungsa[1], Htoo Tint San[1], Boonchoo Sritularak[1,2], Chotima Böttcher[3,4], Eakachai Prompetchara[5,6], Chatchai Chaotham[7,8,*], and Kittisak Likhitwitayawuid[1,*]

1. Department of Pharmacognosy and Pharmaceutical Botany, Faculty of Pharmaceutical Sciences, Chulalongkorn University, Bangkok 10330, Thailand; kittisak.l@chula.ac.th (K.L.)
2. Center of Excellence in Natural Products for Ageing and Chronic Diseases, Faculty of Pharmaceutical Sciences, Chulalongkorn University 10330, Thailand
3. Experimental and Clinical Research Center, a cooperation between the Max Delbrück Center for Molecular Medicine in the Helmholtz Association and Charité – Universitätsmedizin Berlin, Berlin 13125, Germany
4. Max Delbrück Center for Molecular Medicine in the Helmholtz Association (MDC), Berlin 13125, Germany
5. Department of Laboratory Medicine, Faculty of Medicine, Chulalongkorn University, Bangkok 10330, Thailand
6. Center of Excellence in Vaccine Research and Development (Chula Vaccine Research Center), Faculty of Medicine, Chulalongkorn University, Bangkok 10330, Thailand
7. Department of Biochemistry and Microbiology, Faculty of Pharmaceutical Sciences, Chulalongkorn University, Bangkok 10330, Thailand; chatchai.c@chula.ac.th (C.C.)
8. Preclinical Toxicity and Efficacy Assessment of Medicines and Chemicals Research Unit, Faculty of Pharma-ceutical Sciences, Chulalongkorn University, Bangkok 10330, Thailand

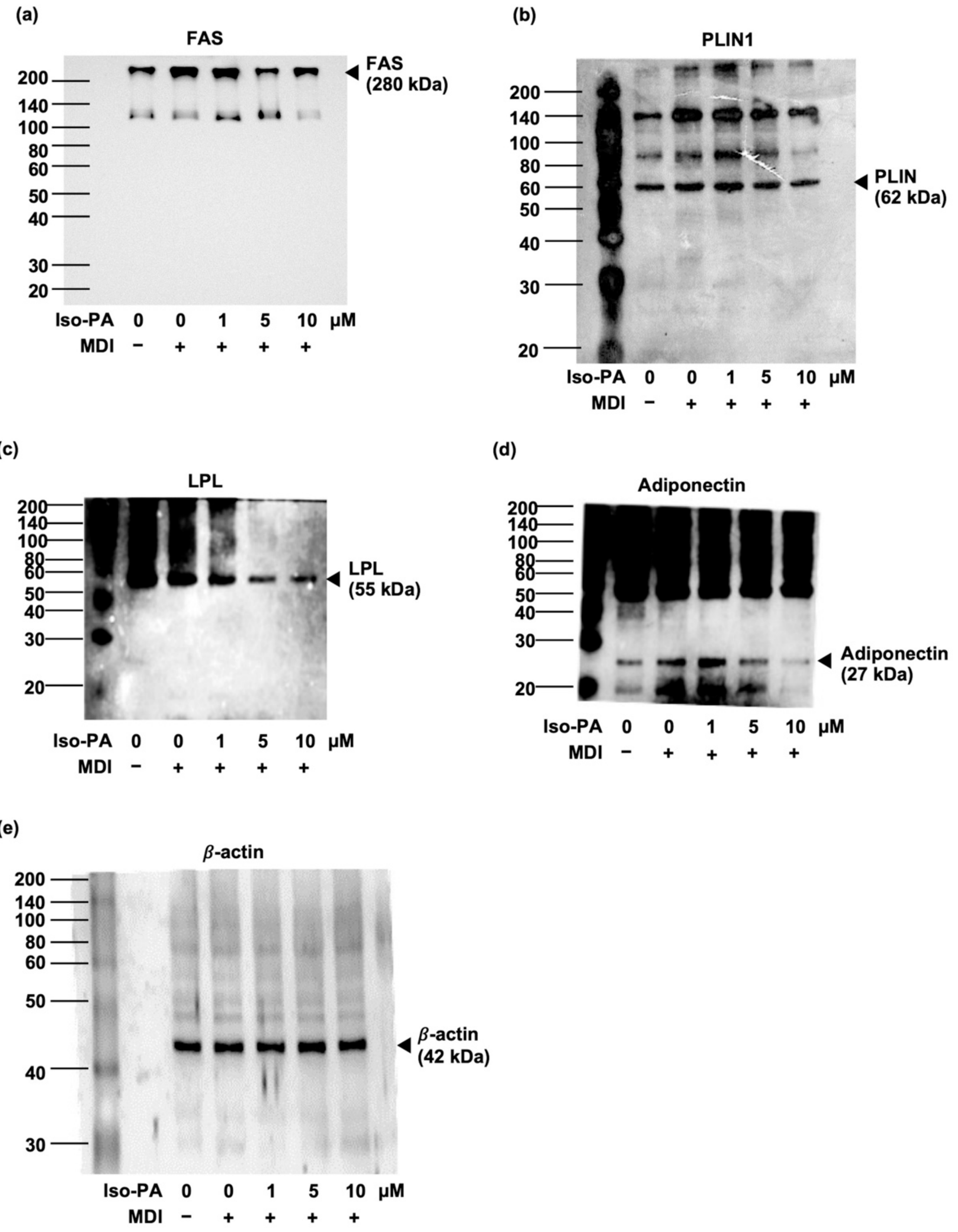

Figure S1. Original western blot images for Figure 3a: Suppression of adipogenic effectors in differentiated 3T3-L1 cells treated with isopanduratin A. Uncropped western blot images show molecular weight markers of (a) FAS, (b) PLIN1, (c) LPL, (d) adiponectin and (e) β-actin as a loading control. The test was present (+) and absent (-) differentiation medium (MDI) containing 0, 1, 5, 10 μM of isopanduratin A (Iso-PA). For experimental details and statistical analysis, see the Experimental and Results sections.

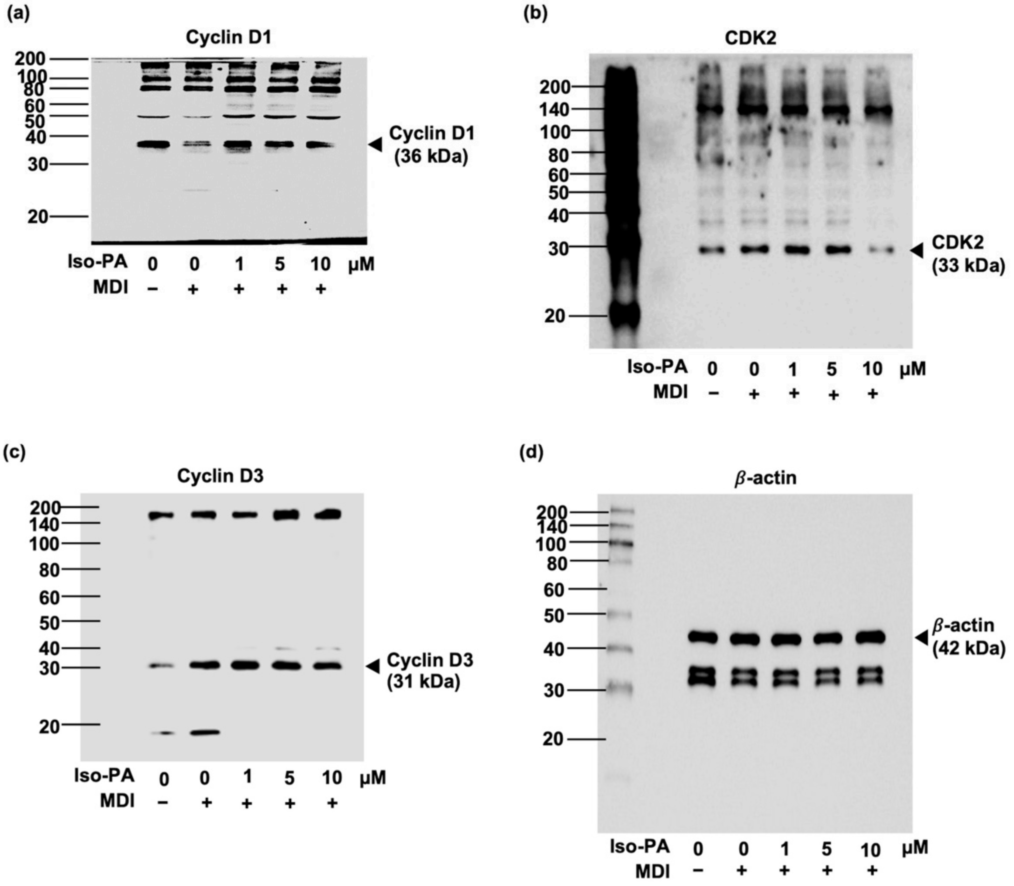

Figure S2. Original western blot images for Figure 4a: Suppression of MCE in differentiated 3T3-L1 cells treated with isopanduratin A. Uncropped western blot images show molecular weight markers of (a) Cyclin D1, (b) CDK2, (c) Cyclin D3 and (d) β-actin as a loading control. The test was present (+) and absent (-) differentiation medium (MDI) containing 0, 1, 5, 10 μM of isopanduratin A (Iso-PA). For experimental details and statistical analysis, see the Experimental and Results sections.

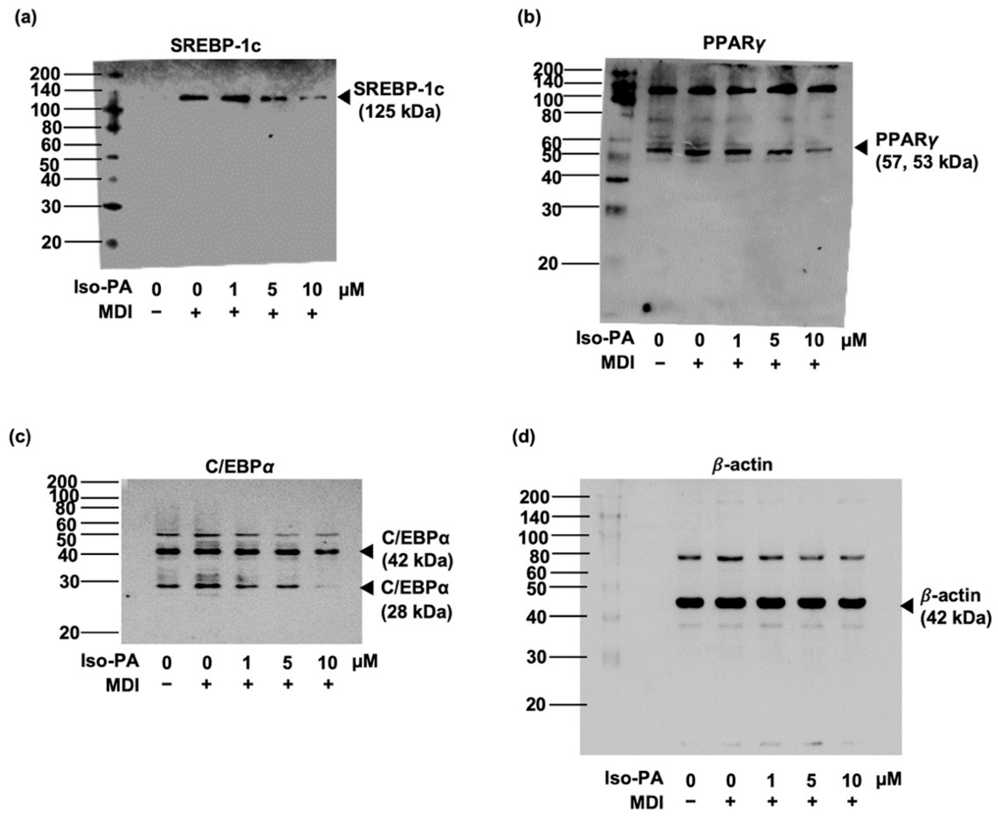

Figure S3. Original western blot images for Figure 5a: Suppression of adipogenic transcription factors in differentiated 3T3-L1 cells treated with isopanduratin A. Uncropped western blot images show molecular weight markers of (a) SREBP-1c, (b) PPARγ, (c) C/EBP*α* and (d) β-actin as a loading control. The test was present (+) and absent (-) differentiation medium (MDI) containing 0, 1, 5, 10 μM of isopanduratin A (Iso-PA). For experimental details and statistical analysis, see the Experimental and Results sections.

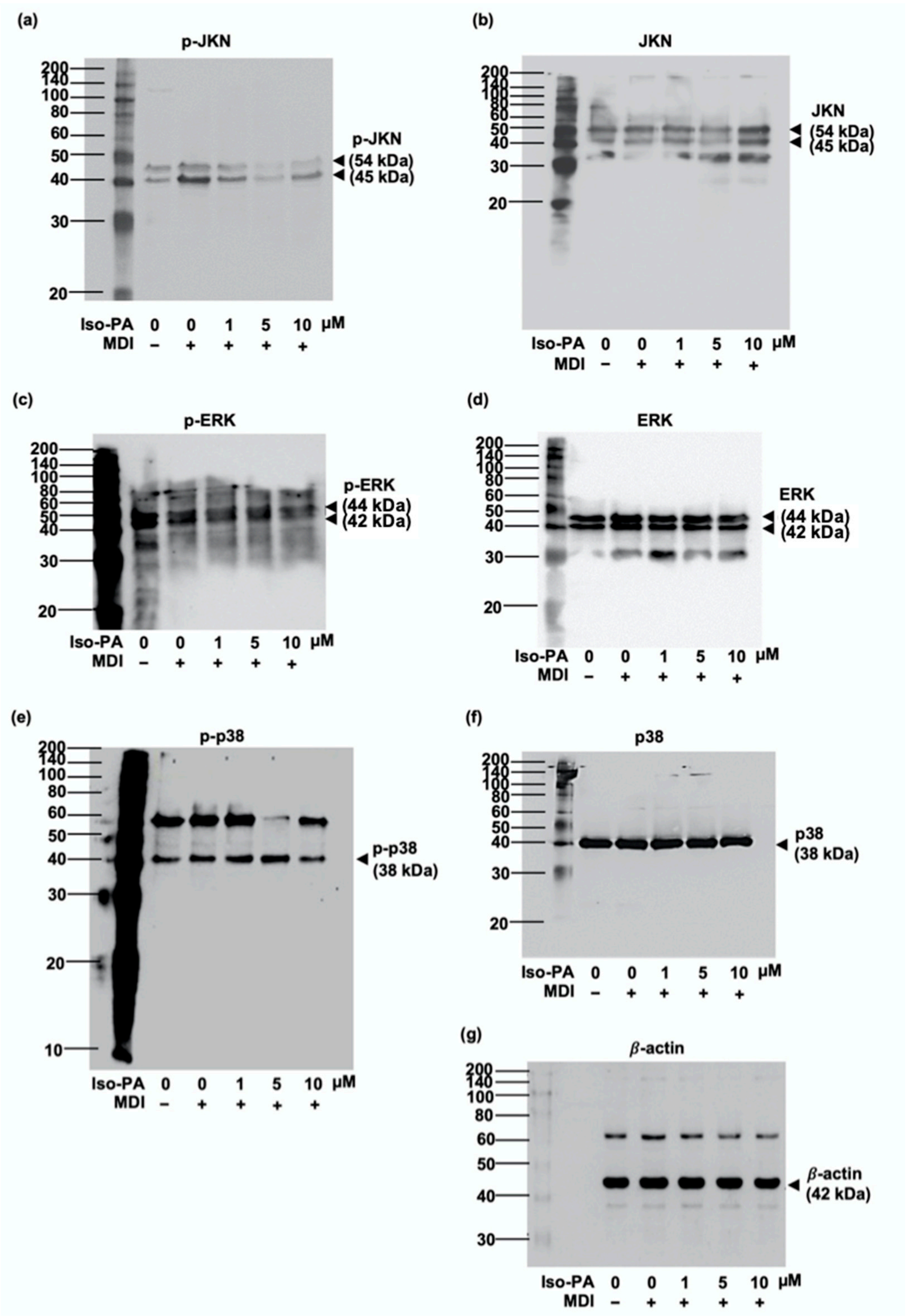

Figure S4. Original western blot images for Figure 6a: Modulation of MAPK signal in differentiated 3T3-L1 cells treated with isopanduratin A. Uncropped western blot images show molecular weight markers of (a) p-JKN, (b) JKN, (c) p-ERK (d) ERK (e) p-p38, (f) p38 and (g) β-actin as a loading control. The test was present (+) and absent (-) differentiation medium (MDI) containing 0, 1, 5, 10 μM of isopanduratin A (Iso-PA). For experimental details and statistical analysis, see the Experimental and Results sections.

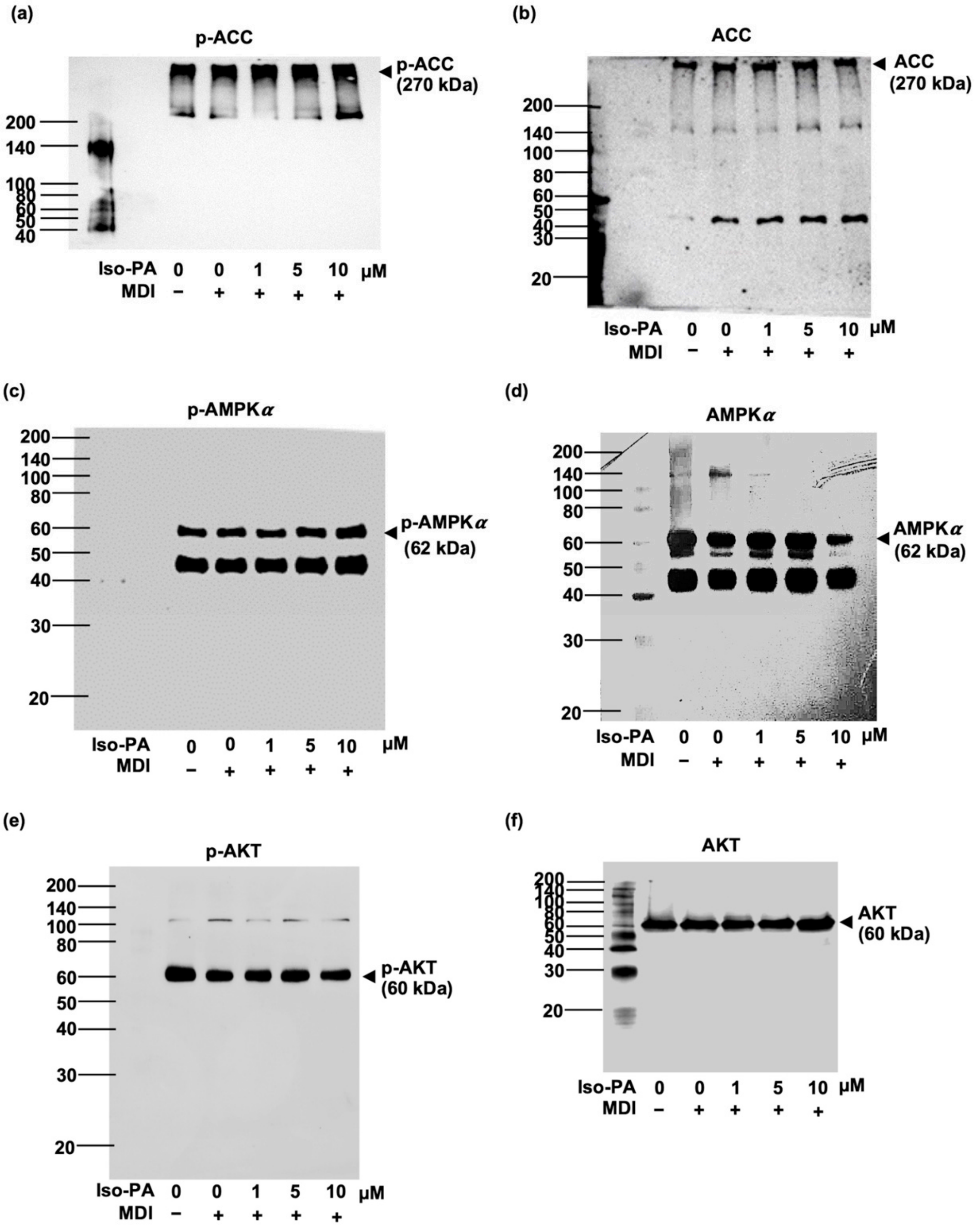

Figure S5 Original western blot images for Figure 7a: Modulation of ATK/AMPK-ACC signal in differentiated 3T3-L1 cells treated with isopanduratin A. Uncropped western blot images show molecular weight markers of (a) p-ACC, (b) ACC, (c) p-AMPKα, (d) AMPKα, (e) p-AKT and (f) AKT. The test was present (+) and absent (-) differentiation medium (MDI) containing 0, 1, 5, 10 μM of isopanduratin A (Iso-PA). For experimental details and statistical analysis, see the Experimental and Results sections.

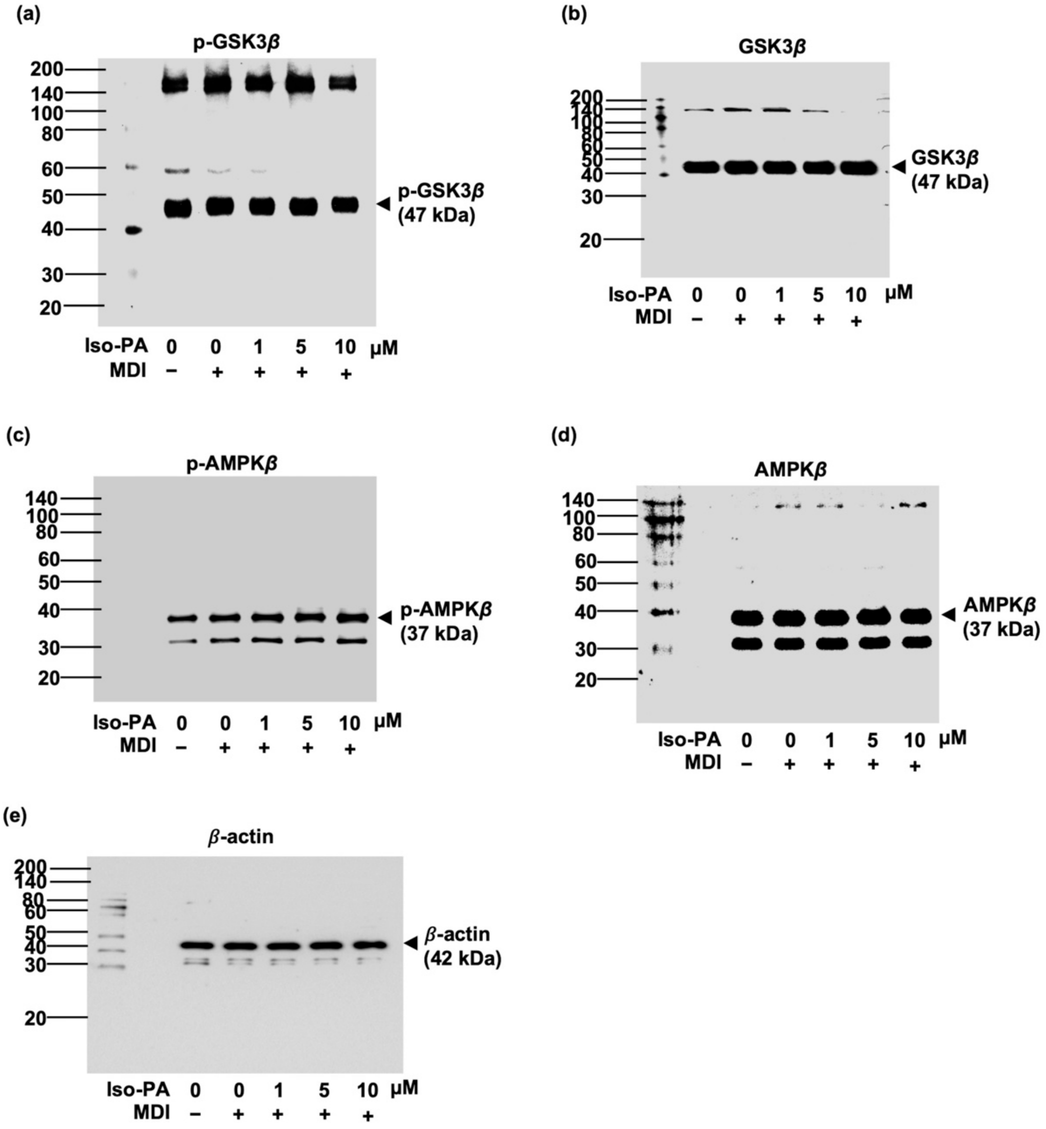

Figure S6 Original western blot images for Figure 7a (continued): Modulation of ATK/AMPK-ACC signal in differentiated 3T3-L1 cells treated with isopanduratin A. Uncropped western blot images show molecular weight markers of (a) p-GSK3β, (b) GSK3β, (c) p-AMPKβ, (d) AMPKβ and (e) β-actin as a loading control. The test was present (+) and absent (-) differentiation medium (MDI) containing 0, 1, 5, 10 μM of isopanduratin A (Iso-PA). For experimental details and statistical analysis, see the Experimental and Results sections.
